# Detection of Structural Variations and Fusion Genes in Breast Cancer Samples Using Third-Generation Sequencing

**DOI:** 10.3389/fcell.2022.854640

**Published:** 2022-04-13

**Authors:** Taobo Hu, Jingjing Li, Mengping Long, Jinbo Wu, Zhen Zhang, Fei Xie, Jin Zhao, Houpu Yang, Qianqian Song, Sheng Lian, Jiandong Shi, Xueyu Guo, Daoli Yuan, Dandan Lang, Guoliang Yu, Baosheng Liang, Xiaohua Zhou, Toyotaka Ishibashi, Xiaodan Fan, Weichuan Yu, Depeng Wang, Yang Wang, I-Feng Peng, Shu Wang

**Affiliations:** ^1^ Department of Breast Surgery, Peking University People’s Hospital, Beijing, China; ^2^ State Key Laboratory of Genetic Engineering, School of Life Sciences and Human Phenome Institute, Fudan University, Shanghai, China; ^3^ GrandOmics Inc., Beijing, China; ^4^ Department of Pathology, Peking University Cancer Hospital, Beijing, China; ^5^ Department of Statistics, The Chinese University of Hong Kong, Sha Tin, China; ^6^ Department of Biostatistics, School of Public Health, Peking University, Beijing, China; ^7^ Department of Electronic and Computer Engineering, Hong Kong University of Science and Technology, Kowloon, Hong Kong SAR, China; ^8^ Division of Life Science, Hong Kong University of Science and Technology, Kowloon, Hong Kong SAR, China

**Keywords:** long-read sequencing, breast cancer, structural variation, fusion gene, sequencing panel

## Abstract

**Background:** Structural variations (SVs) are common genetic alterations in the human genome that could cause different phenotypes and diseases, including cancer. However, the detection of structural variations using the second-generation sequencing was limited by its short read length, which restrained our understanding of structural variations.

**Methods:** In this study, we developed a 28-gene panel for long-read sequencing and employed it to Oxford Nanopore Technologies and Pacific Biosciences platforms. We analyzed structural variations in the 28 breast cancer-related genes through long-read genomic and transcriptomic sequencing of tumor, para-tumor, and blood samples in 19 breast cancer patients.

**Results:** Our results showed that some somatic SVs were recurring among the selected genes, though the majority of them occurred in the non-exonic region. We found evidence supporting the existence of hotspot regions for SVs, which extended our previous understanding that they exist only for single nucleotide variations.

**Conclusion:** In conclusion, we employed long-read genomic and transcriptomic sequencing to identify SVs from breast cancer patients and proved that this approach holds great potential in clinical application.

## Background

Breast cancer is the most common malignancy in women. Genome instability is a critical molecular characteristic of breast cancer, whereas structural variation directly manifests genome instability (Duijf et al., 2019). Structural variations (SVs), including insertion, deletion, duplication, inversion, and translocation, affect nucleotides on a much larger scale over single nucleotide variations (SNVs) (Sudmant et al., 2015). SVs are common variations in the general population, as shown by the 1,000 genome project (Iafrate et al., 2004; Sebat et al., 2004), where specific variations are known to be responsible for developing a number of genetic diseases and cancers (Feuk et al., 2006; Sharp et al., 2006; Alkan et al., 2011; Li et al., 2020). Previous studies of structural variation influence on gene structure and expression have significantly deepened our understanding of tumorigenesis (Hollox et al., 2021). Many oncogenes have been proven to be the products of chromosomal translocations and can be served as therapeutic targets. However, it remains challenging to identify SVs in the cancer genome due to the limitation of the next-generation sequencing (NGS), i.e., short read-length and sequence preference in PCR, which hinder NGS from detecting complex SVs. Moreover, algorithms trying to identify SVs from NGS data of short read-length showed a high false-negative rate (Sobczak and Krzyzosiak, 2002). Third-generation sequencing (TGS) techniques, including Single-Molecule Real-Time (SMRT) sequencing of Pacific Biosciences (PacBio) and the Nanopore long-read single-molecule sequencing of Oxford Nanopore Technologies (ONT), have shown higher sensitivity and specificity in structural variation detection and have been applied in tumor research including breast cancer research (Nattestad et al., 2018; Vasan et al., 2019; Aganezov et al., 2020).

Even though SVs of breast cancer in SKBR-3 cell line and patient-derived organoids have been widely studied (Zhuang et al., 2021), more proof is needed to illustrate the relationship between SVs and cancer. Nevertheless, the emerging TGS technologies with long-read capability have demonstrated their strengths in cancer study, which allows us to analyze the haploid genome at unprecedented precision. They could provide valuable insights into precision medicine, as in the case of double *in-cis* PIK3CA mutations showing high sensitivity for alpesilib (Vasan et al., 2019).

In this study, we aim to accurately detect DNA structural variations of a 28-gene panel in breast cancer tissue, matched by para-tumor tissues and blood samples via both ONT and PacBio TGS platforms. To the best of our knowledge, this study was the first to comprehensively analyze structural variation in breast cancer tissue directly via multiple TGS technologies.

## Methods

### Ethical Approval

The study was approved by the Peking University People’s Hospital ethics committee (Reference number 2021PHB227-001).

### DNA Extraction

Genomic DNA was extracted from the frozen tissue/blood specimens using the standard phenol/chloroform extraction protocol. Briefly, the tissue specimens were fully ground with liquid nitrogen. For blood, 1 ml whole-blood samples were added with an equal amount of ice-cold cell lysis buffer (1.28 M sucrose, 40 mM Tris hydrochloride, 20 mM MgCl_2_, 4% Triton X-100 [pH 7.5]), and three volumes of ice-cold distilled water. This mixture was incubated for 10 min on ice, and the nuclear pellet was collected by centrifugation (6,000 rpm, 5 min, 4°C). The nuclei both from tissue and blood samples were suspended in extraction buffer (1 M sodium chloride, 100 mM Tris, and 50 mM EDTA, buffered at pH 8.0) containing 2% sodium dodecyl sulfate (SDS) and proteinase K (2 mg/ml final concentration). The suspended nuclei were incubated at 56°C for 2 h, extracted with phenol-chloroform-isoamyl alcohol (28:24:1 by volume), one more time with chloroform-isoamyl alcohol (24:1 by volume), and precipitated with 0.7 volume of isopropyl alcohol at −20°C for 40 min. The DNA precipitates were washed in ice-cold 80% ethanol twice, collected by centrifugation (12,000 rpm, 15 min, 4°C), dried under vacuum, and finally resuspended in 100 ul of EB (10 mM Tris hydrochloride [pH 8.0]) (#19086, Qiagen). The quantity and quality of DNA samples were measured by NanoDrop One (ND-ONE-W, Thermo Fisher Scientific Inc.) and on 1% agarose gel electrophoresis.

### Target Regions Capturing and Sequencing

DNA probes of 120 bases were designed to cover full-length genes of interest as a custom-made DNA-Cap Panel and were synthesized by Boke Biotechnologies (Wuxi, Jiangsu, China). During the design of the probes, the Repeat Masker dataset was used to remove probes corresponding to repetitive sequences in the human genome. Capture and enrichment of regions of interest were performed following the manufacturer’s protocol. Briefly, 3 ugs genomic DNA was sheared to around 5–6 kb fragments by a g-TUBE (#520079, Covaris, Woburn, MA, United States) centrifugation (15,000 g, 2 min, twice). End-repair and dA-tailing of DNA fragments according to protocol recommendations were performed using the Ultra II End Prep module (#E7546, NEB) through pre-capture amplification. Targeted sequence capture was conducted by pooling indexed PCR products and hybridizing them with custom-made probes. Captured DNA fragments were amplified by PCR again using universal primer. After purification, the prepared target DNA was sequenced using the Pacific Biosciences (PacBio, Menlo Park, CA, United States) SMRT sequencing technology according to protocol recommendations. The PacBio SMRT Bell™ sequencing library was constructed using a SMRTbell Express Template Prep Kit 2.0 (#100-938-900, PacBio). Finally, sequencing was performed on the PacBio Sequel II platform according to the manufacturer’s instructions.

### Data Quality Control and Detection/Annotation of SVs

Raw sequencing data (also called raw polymerase reads) were first tested in a standard quality control protocol by using the SMRTlink 8.0 (PacBio) to remove low-quality reads and adapters resulting in subreads. The minimum polymerase reads accuracy was 0.75. The read quality (RQ) was marked as 0.8 if passed the quality control or as 0 if failed in the filtering. Subreads were obtained by the above filtering. Circular Consensus Sequence (CCS) was used to get CCS reads, and Lima was used for barcode splitting. PBMarkDUP (PacBio) was used to remove potential copies in CCS reads, and PBMM2 (PacBio) was used to compare CCS reads to the reference genome hg38. PBSV (V9.0, https://www.pacb.com/support/software-downloads/) was used to detect SVs, and DeepVariant (Poplin et al., 2018) (V1.0.0, https://github.com/google/deepvariant) was used to detect SNP and InDel. Detected mutations were be annotated by Annovar (Wang et al., 2010) (http://nar.oxfordjournals.org/content/38/16/e164) if the following criteria have been met. For SVs, 1) the number of supported reads with mutations ≥2; 2) mutation frequency among tumor samples ≥0.1; 3) mutation frequency = 0 in reference, and 4) screening mutations at interested regions. For SNP and InDel, 1) number of reads covering mutation sites ≥5, 2) the number of reads with mutations ≥2, 3) mutation frequency among samples ≥0.05; 4) the number of reads covering mutation sites ≥ in reference control ≥0; 5) the frequency ratio between reference and tumor samples <0.143, and 6) screening mutations at interested regions.

### RNA Sample Preparation, cDNA Library Construction, and Sequencing

Total RNA from each tissue sample was extracted using the RNeasy Plus Mini Kit (Qiagen, Germany). The RNA purity was checked using the NanoDrop™ One (Thermo Fisher Scientific, United States). RNA degradation and contamination were monitored using 1% agarose gels. The RNA concentration was measured using the Qubit® RNA Assay Kit in the Qubit® 3.0 Fluorometer (Life Technologies, CA, United States). The RNA integrity was assessed using the RNA Nano 6000 Assay Kit of the Bioanalyzer 2,100 system (Agilent Technologies, CA, United States). The RNA quality criteria for the RNA samples was RNA Integrity Number (RIN) > 8.0 and 2.0 < OD 260/280 < 2.2. Qualified RNAs were used for Nanopore library preparation. First, reverse transcription of qualified RNA, PCR amplification, and adapter ligation were performed using the library preparation kit SQK- PCS109 (Oxford Nanopore Technologies) following the recommended protocol. Then prepared libraries were sequenced on a Nanopore PromethION platform using flowcell R9.4.1.

### Preprocessing of Sequencing Reads and Genome Mapping

For the raw sequencing reads, reads of which quality score is lower than seven or length is shorter than 200 bp were discarded using quality control tool Nanofilt (De Coster et al., 2018) (https://github.com/wdecoster/nanofilt). Then full-length reads were identified and oriented from sequencing reads by the pychopper tool (https://github.com/nanoporetech/pychopper) with default parameters. Then full-length reads were aligned to the hg38 reference genome using minimap2 (Li, 2018) (-ax splice -uf -junc-bed). Genome mapping results of full-length reads were visualized using the Integrative Genome Viewer (Robinson et al., 2011).

### Prediction of Coding Sequences and Fusion Transcript Identification

Prediction of coding sequences and protein sequence was performed in all novel isoforms using the ANGEL software (Shimizu et al., 2006) (https://github.com/PacificBiosciences/ANGEL). Fusion transcripts were identified using fusion_finder.py from software cDNA_Cupcake (https://github.com/Magdoll/cDNA_Cupcake). Specifically, an identified fusion transcript must meet the following criteria: 1) fusion transcripts map to two or more loci in the genome; 2) each mapped locus must align with at least 95% identity and at least 5% coverage; 3) total aligned coverage of the fusion transcript must be above 99%; 4) each mapped locus must be at least 10 kb apart.

## Results

### Target Regions Capturing and Coverage

**FIGURE 1:**
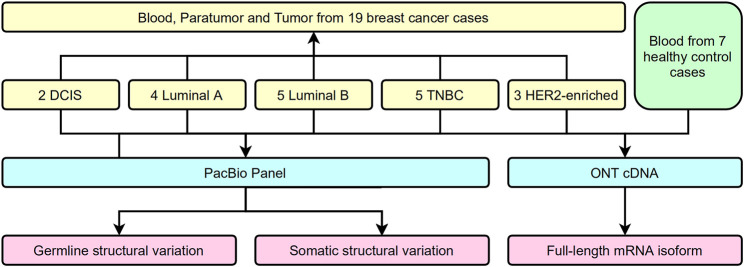
Flow chart of study design.

Several approaches have been used to examine the genomic and transcriptional signatures in breast cancer patients. We recruited 19 breast cancer patients and seven control cases in this long-read study during 2019–2020 (Figure 1). All experimental designs and procedures abide by the regulations from the Institutional Review Board of Peking University People’s Hospital. Multiple subtypes of breast cancer were selected as research subjects in this study, including four invasive subtypes (Luminal A, Luminal B, HER-2 enriched, and Triple Negative Breast Cancer (TNBC) cases previously classified by immunohistochemical staining) and Ductal Carcinoma *in situ* (DCIS) cases (Table 1). Three sets of samples (blood, para-tumor, and tumor) were obtained from all patients. Long-read DNA and RNA information was obtained for a 28-gene panel using the PacBio platform and the ONT full-length whole transcriptome platform, respectively (refer to methods). In addition, blood samples from 7 healthy control donors were processed with the same procedures (Figure 1).

**TABLE 1:** Clinicopathological features of breast cancer patients and healthy controls recruited.

Classification	Patient #	Tumor Size (cm)	Lymph Node Metastasis	Ki-67	Sample Inclusion		
					B	P	T
DCIS	RM65	3*2	0	10%	DNA	DNA & RNA	DNA
	RM80	0.5	0	10%	DNA	DNA	DNA & RNA
HER2	RM62	3*1	0	30%	DNA	—	DNA & RNA
	RM63	3*1.5	0	15%	—	DNA & RNA	DNA & RNA
	RM71	2.5*2	2	30%	DNA	DNA	DNA & RNA
Luminal A	RM66	1.7*1.4	0	10%	—	DNA & RNA	RNA
	RM70	2.4*2	3	10%	DNA	DNA	DNA & RNA
	RM74	4.9*4*2.4	1	10%	DNA	DNA & RNA	DNA & RNA
	RM77	1.7*1.2	0	5%	—	DNA	RNA
Luminal B	RM72	2.5*2.5*1.8	2	40%	DNA	DNA	DNA & RNA
	RM73	3*2.8	6	50%	DNA	DNA & RNA	DNA & RNA
	RM76	9.5*7.5*1.8	1	20%	DNA	DNA & RNA	DNA & RNA
	RM78	0.6	0	40%	DNA	DNA	DNA & RNA
	RM79	4.3*3.7*2.7	2	20%	DNA	—	DNA & RNA
TNBC	RM64	2.7*2.2	0	70%	DNA	DNA & RNA	DNA & RNA
	RM67	1.6*1.5	3	70%	DNA	DNA & RNA	DNA & RNA
	RM68	2.8*2*1.9	1	90%	—	DNA & RNA	RNA
	RM69	1.5*1*1	0	70%	DNA	DNA & RNA	DNA & RNA
	RM75	2.5*2	0	20%	DNA	DNA	DNA & RNA
Healthy Control	RMH3				DNA		
	RMH7				DNA		
	RMH9				DNA		
	RMH12				DNA		
	RMH15				DNA		
	RMH20				DNA		
	RMH25				DNA		

**FIGURE 2:**
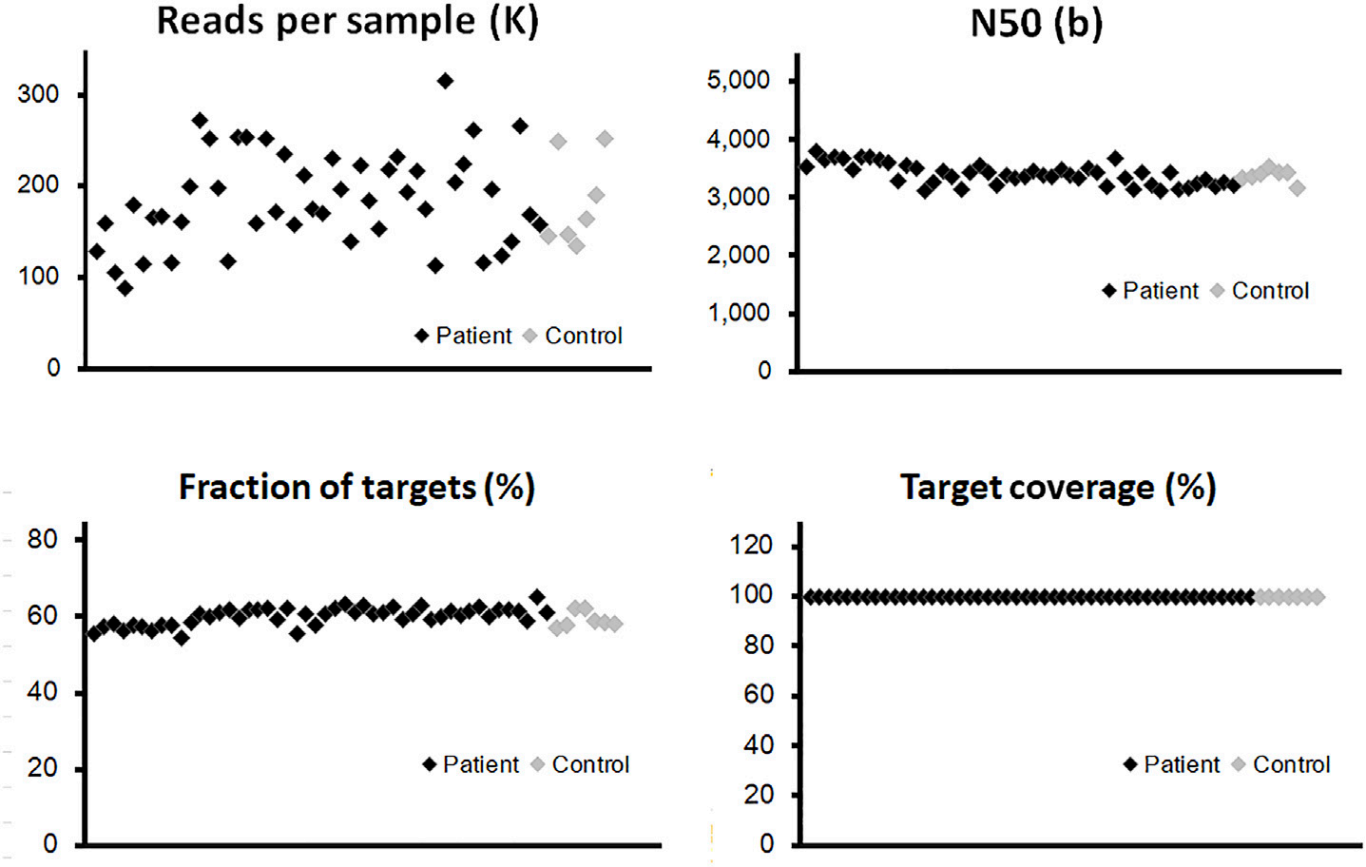
Quality control of long-read sequencing for the panel of 28 genes. The probes were designed to cover the whole genome regions of all panel genes, which are around 5M-base coverage of detection. The vertical axis of each point illustrated the quantitative information from individual blood sample, para-tumor tissue, or tumor tissue. The effective read numbers were around 100–300 kb per sample, and the N50s were around 3,000–4,000. No obvious differences could be detected between patient and control groups (black and gray points, respectively). After the alignment process, the fraction of targets among different samples was around 55% with a slightly fluctuation. The coverage of the target region was above 99% in all tested samples.

By the combination of a full-length panel approach and long-read sequencing tools, it was possible to explore not only SNPs but also most SVs within these genes, regardless of their locations at either exons or introns. The panel in our study focuses on two gene types: twenty genes associated with a high risk of breast cancer and also participated in homologous recombination repair (HRR) (Breast Cancer Association et al., 2021; Hu et al., 2021; Yadav et al., 2021), and eight genes involved in the precision medicine during breast cancer treatment (Harbeck et al., 2019; Sparano et al., 2019; Waks and Winer, 2019) (Supplementary Table S1). Probes were designed to cover the whole genome regions of these genes, which are about 5 M bases. Our results are shown in Figure 2; Supplementary Table S2 summarized some essential characteristics of this panel plus a long-read approach: sufficient depth of sequencing, long reads (N50 is around 3,500 bases), and high target coverage (>99.5%). There were no significant differences in these essential characteristics among the three types of samples and no apparent disparity between samples from patients and healthy controls (Figure 2; Supplementary Table S2).

### Analysis of Germline SVs in Breast Cancer Patients

**FIGURE 3:**
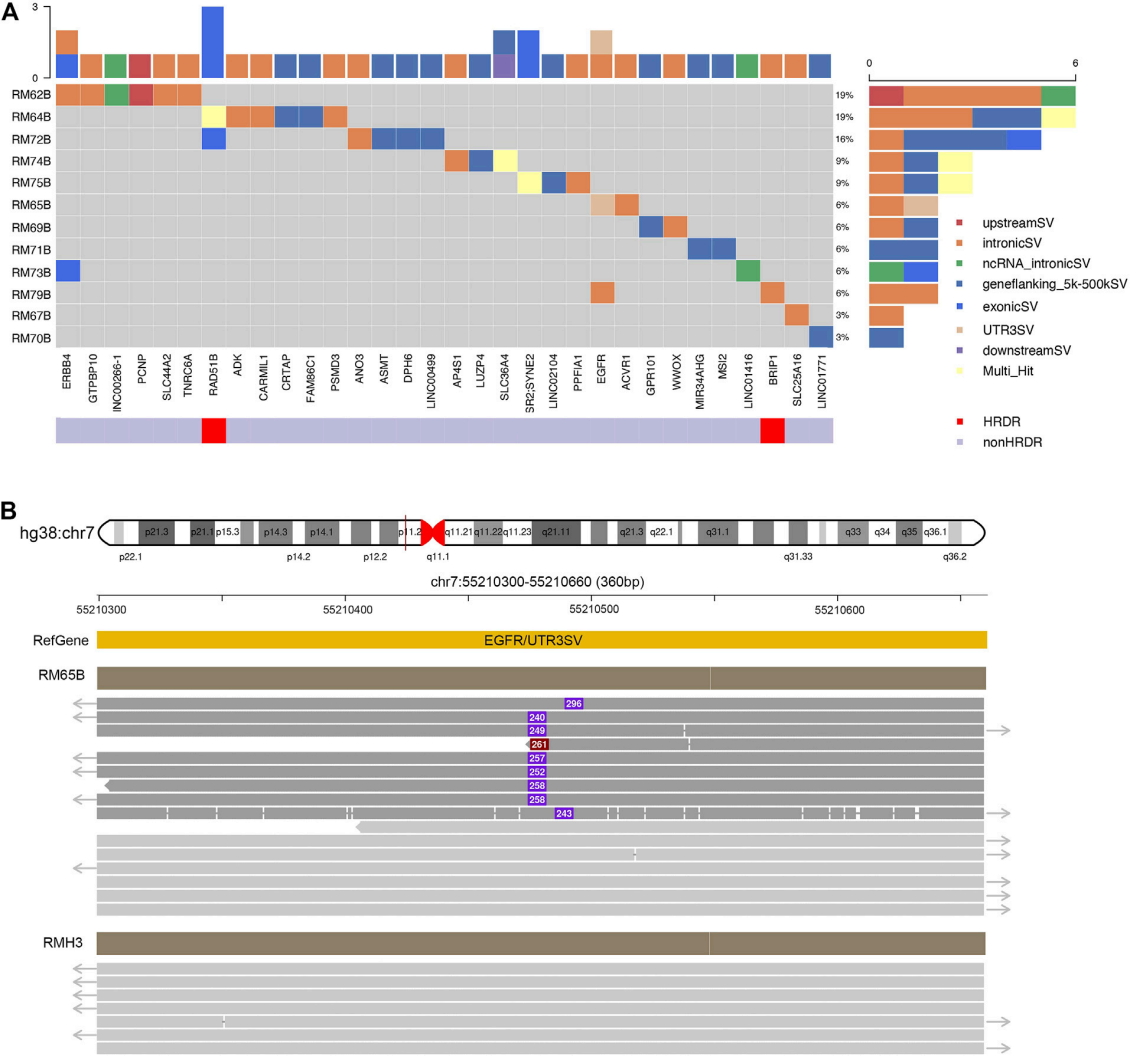
Excessive germline structural variants occurred in non-exonic regions in breast cancer patients. **(A)** Summary of germline SVs in specific genes and patients. Individual patients’ blood samples (starting with “RM” labeling, plus patient number, and end with B for blood samples) were examined against to seven healthy samples as control. The calling of SVs would be classified to one of the following categories: exons, introns, upstream or downstream regions, un-translated regions (UTRs) at 3′ or 5′ side, flanking regions of genes within 2 kilo-bases, or multiple-hit sites. The scales in the top and right insets illustrated the cumulative numbers of SVs in particular genes and patients, respectively. Most SVs were located at non-exonic regions. **(B)** SVs identified in EGFR. In RM65B, an insertion (∼280 bp) was identified at the UTR 3′ region of EGFR genes. The dark red solid line in hg38:chr7 pointed to a 360-bp region as expanded below. Representative reads from RM65B and RMH3 (control) were aligned accordingly. The purple boxes and inside numbers showed the locations and sizes of this insertion in individual reads. Such insertion was identified only in a part of reads in RM65B (24/49) but not in RMH3 (0/83).

In our panel study, germline SVs were detected in the blood sample of 12 patients (12/19, 63%) against the healthy controls (Figure 3). The number of SV carried by a single patient varies from one to six (left subset, Figure 3A). Based on their locations, these SVs could be classified into exons, introns, upstream or downstream regions, untranslated regions (UTRs) at 3′ or 5’ side, flanking regions of genes within two kilo-bases, or multiple-hit sites, which means more than one of the aforementioned categories. Only a few SVs were found at exonic regions (6/33, bright blue blocks, upper inset in Figure 3A), which agrees with previous studies (Sakamoto et al., 2020). From another perspective, SVs could be found in HRD genes like *RAD51B* and *BRIP1* or treatment-related genes like *ERBB4* and *EGFR4*. The distribution of germline SVs in these genes was sporadic, and no apparent high-frequency genes were counted, presumably due to the relatively small number of samples.

Notably, our long-read plus full-length-gene approach allowed us to detect SVs at locations that were hardly noticed by the conventional short-read techniques (Figure 3B). For instance, an about 250-base insertion at 3’ UTR of EGFR was seen in patient RM65B, but not in healthy control RMH3. This UTR region is close to the centromere of Chromosome 7 and contains many TA repeats. Meanwhile, among individual reads, the locations of this insertion and its size are slightly different, as shown in Figure 3B, which further demonstrates the complexity of this mutation site.

### Potential Hotspot of Somatic SVs Revealed in Tumor Tissue

**FIGURE 4:**
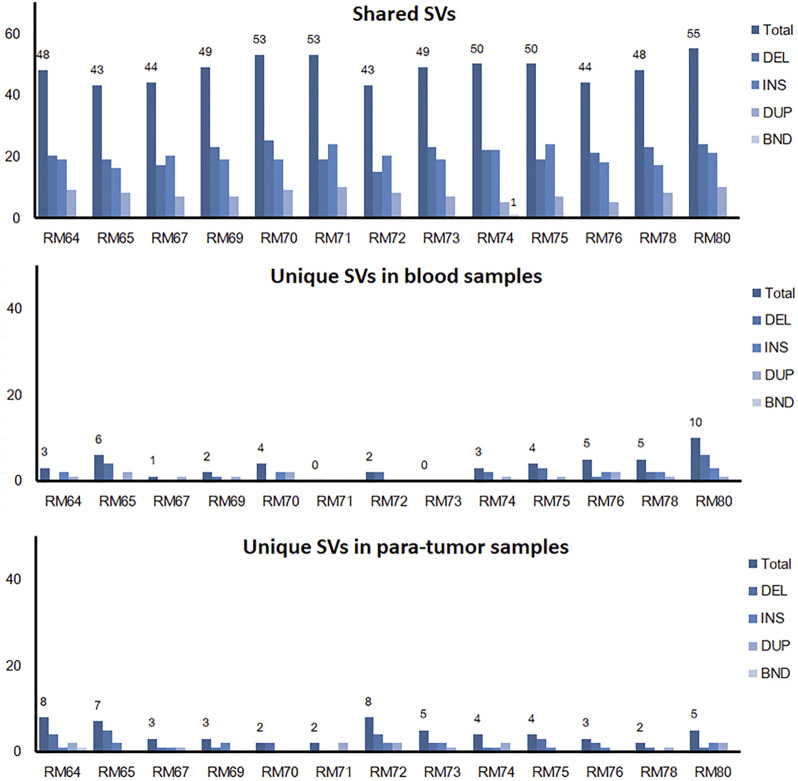
Shared and unique SVs in para-tumor tissue and blood samples. A comparison of shared and unique SVs between two kinds of samples. Numbers above individual bars showed the number of total SVs. DEL, deletion; INS, insertion; DUP, duplication; and BND, Breakpoint notation. Most SVs were found in both tissues, while a few unique SVs were only observed in one of the tissues.

**FIGURE 5:**
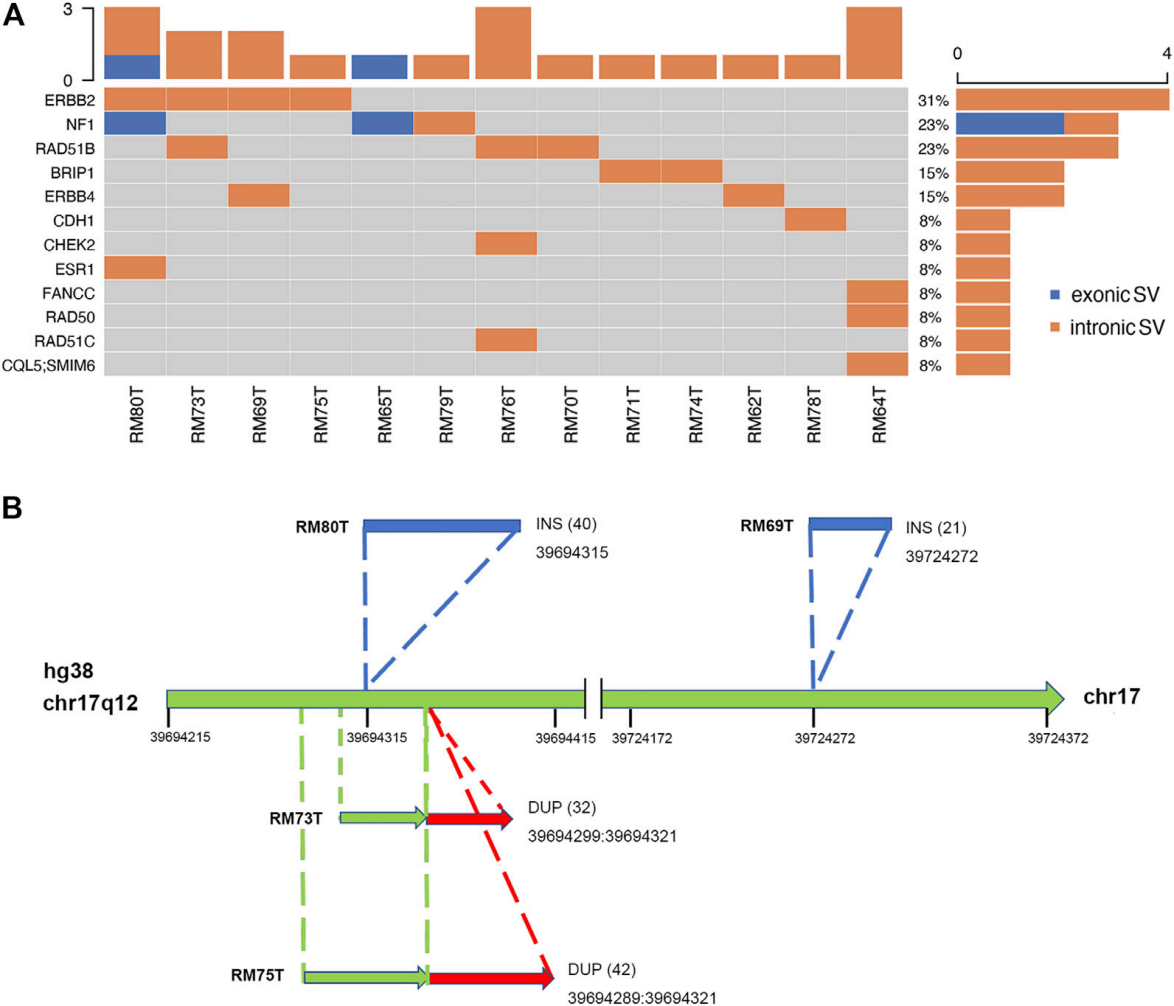
Excessive somatic SVs in non-exonic region. **(A)** A summary of somatic SVs in particular genes and patients. Patients’ tumor samples were examined against to their blood samples as control. SVs are sorted into exonic and intronic types based on their locations. Similarly, most SVs were found at non-exonic regions. Among thirteen patients, four of them had been identified to carry SVs at ERBB2. **(B)** Hotspot of SVs in ERBB2. There were two INS SVs (shown in blue) and two DUP SVs (red) found in ERBB2. Numbers inside parentheses indicated the sizes of SVs. A conserved region with SV occurrence was exposed among three independent patients (RM80T, RM73T, and RM75T).

The somatic SVs could be identified by annotating the unique SVs in tumor tissues against those in either blood samples or para-tumor tissues. When comparing SVs detected from para-tumor samples with that from matching blood samples, it was found that most of them were shared by both control samples (43–55 per patient, the upper plot in Figure 4, see also Supplementary Table S3), implying that these common SVs are background germline variants. Meanwhile, the existence of unique SVs in para-tumor samples (0–10, middle plot) and blood samples (2–8, bottom plot) was possibly caused by loss of heterozygosity (LOH). It indicated that the para-tumor tissue, which was histologically normal, had already been genetically altered in terms of SVs. This is consistent with findings from SNV studies (Hu et al., 2018). Our study used blood samples as a reference for tumor tissues to find cancer-driven SVs. The somatic SVs in tumor tissue affecting the 28 breast cancer-related genes were identified and displayed in Figure 5 ; Supplementary Table S4. Our results showed that each patient carried none or only a few somatic SVs (0–3, 13 out of 19 patients had SVs ≥1, upper inset in Figure 5A) in this 28-gene panel study. Meanwhile, somatic SVs were detected in 12 out of 28 genes (12/28, 43%, right inset of Figure 5A). SVs had been classified into exonic and intronic according to their locations. In consistent with previous studies, most SVs were identified within the intronic region (Tuzun et al., 2005). Among the 12 genes, *ERBB2* had the highest SV frequency, which was detected in 4 patients and being all intronic, followed by *NF1* and *RAD51B*. Figure 5B summarized the four cases of SVs in *ERBB2*: two insertions and two duplications. Noteworthy, three of these cases have starting sites close to each other, resulting in a certain degree of overlap among the following sequences (RM73T, RM75T, and RM80T). These patients were clinically divided into three groups (Luminal B, TNBC, and DCIS). As far as we know, this region was AT-rich and had not been reported to cause disease. However, the relative enrichment of somatic SVs at the same site in *ERBB2* (3 out of 19 independent patients) suggests that this is an interest area in breast cancer and needs further validation with more samples.

### Full-Length Transcriptome Analysis of Tumor and Para-tumor

**FIGURE 6:**
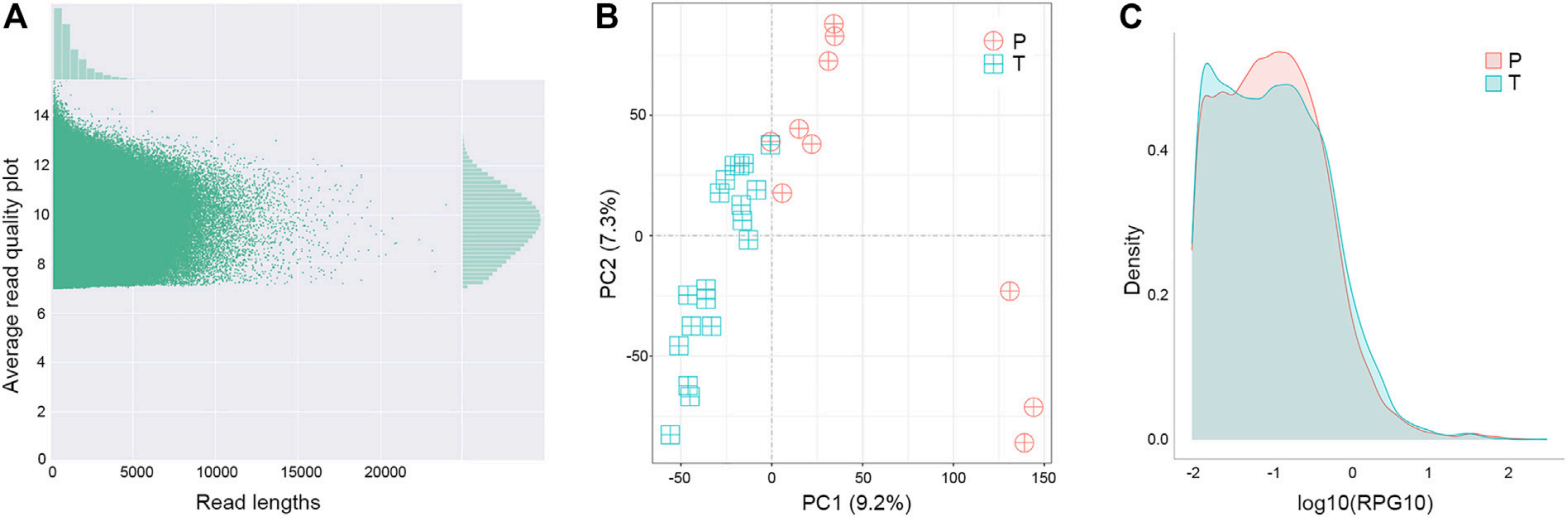
Distinguishable transcriptomes of tumor samples from others in long-read sequence. **(A)** Quality control of transcriptome analysis. Transcriptome was built-up based on their cDNA library construction followed by ONT nanopore sequencing. Plot showed the quality of individual data points (main figure) as well as their distributions (top and left insets). Data points below 7 in Average Read Quality (i.e., lower than 85% accuracy from ONT manual instruction) were excluded from analysis. **(B)** Principal component (PC) analyses. Each symbol represented one clinical sample from para-tumor tissues (P) and tumor tissues (T). Clearly the majority of samples were quite distinguishable from ones from the other group. **(C)** Density of reads per gene10 (RPG10K) plot. Differential patterns from different types of samples revealed that tumor tissues had smaller reads per genes than para-tumor tissues.

Changes at the transcriptional level could provide support and direct evidence for genomic mutations. The cDNA of para-tumor and tumor were sequenced using the Nanopore PromethION platform to get the full-length transcriptome data. Data points below 7 in Read Quality (accuracy lower than 85%) were excluded, and the valid points were scattered based on their length in Figure 6A. Our results showed that the average read quality was about 10, and the mean and median for read length were 1.3 k and 1.9 k base pairs, respectively. Principal component analysis revealed that the para-tumor and tumor tissues could be efficiently distinguished based on their transcriptomic data (red and green dots, respectively, Figure 6B). The density plot of reads per gene per 10,000 reads (RPG10K) showed that the reads per gene of tumor tissues is apparently shorter than that of the para-tumor tissues, but this conclusion can’t be drawn at this stage due to the small sample size (Figure 6C). With all mentioned characteristics taken together, it suggests that long-read sequencing on transcriptome could potentially be a good candidate technique for diagnostic application in the future.

### Gene Fusions With Both Genomic and Transcriptomic Evidence

**FIGURE 7:**
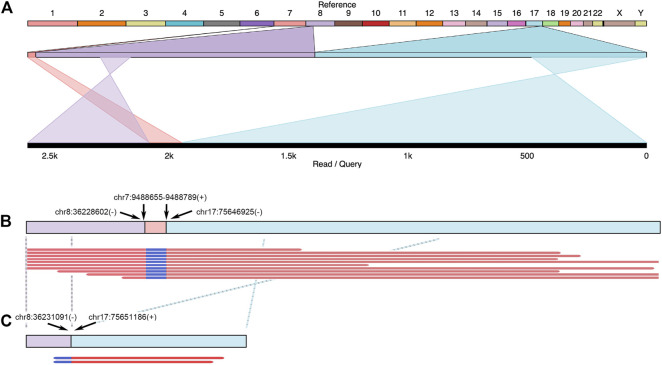
Example of fusion genes and transcripts observed in patient tumor tissues. In Patient RM64, several structural variants (including DEL and BND) were identified at FANCC, RAD50, and RECQL5 regions. A three-segment fusion gene observed at RECQL5 was illustrated. **(A)** Top line: reference genome from hg38. Numbers and letters indicated individual chromosomes. Middle line: expansion of sequenced regions from chromosomes 7, 8, and 17. Bottom line: Illustration of one genomic structure in RM64T samples, containing regions from chromosomes 8, 7, and 17. Crossed projection lines from middle to bottom lines represented reversions occurred during the fusion process. **(B)** Top line: a plot of this fusion gene at genomic DNA. Breakpoint notation locations were labeled by arrows. Plus and minus symbols showed forward and reverse directions, respectively. Bottom lines: representative data selected from individual reads. **(C)** Transcripts observed in this area. Top line: a cartoon demonstration of corresponding mRNA. Crossed dash lines showed a reversion observed after a comparison to reference. Bottom lines: two reads continuously from chr8 to chr17 were shown. The chr7 segment in this fusion gene did not have detectable mRNA reads.

The accumulation of fusion genes is one of the patterns commonly found in tumor tissues (Matsushige et al., 2019). However, how the fusion genes contribute to or are formed during cancer progression is barely documented. Due to their long-read sequencing characteristics, PacBio, and ONT platforms might benefit to studying fusion genes. In a total of 19 cases, we reported that there were seven fusion genes observed in six patients (Table 2). One case found in RM64 showed that a fusion gene at *RECQL5* in Chromosome 17 contained two other segments from Chromosomes 8 and 7 (Figure 7A), showing a particular case of a three-segment fusion gene. The confidence of this fusion gene is supported by its high coverage (depth >30X, Figure 7B) of reads obtained by the high fidelity PacBio HiFi platform. In addition, transcripts of this fusion gene were also obtained from the ONT Platform (Figure 7C). It appeared that a certain degree of alternative splicing was processed (indicated by dash lines between Figures 7B,C), resulting in a missing of Chromosome-7 segment as well as shorter length in transcripts.

**TABLE 2:** Fusion genes detected in tumor samples.

Sample	Chr	Start	SV type	SV ID	Gene	Location
RM64	chr17	75646925	BND	pbsv.BND.chr17:75646925-chr7:9488789_1	RECQL5; SMIM6	intronic
RM64	chr9	95119452	BND	pbsv.BND.chr9:95119452-chr19:36870553_1	FANCC	intronic
RM71	chr17	61860680	BND	pbsv.BND.chr17:61860680-chr17:72564876_1	BRIP1	intronic
RM76	chr17	58714578	BND	pbsv.BND.chr17:58714578-chr17:57731280_1	RAD51C	intronic
RM78	chr16	68793666	BND	pbsv.BND.chr16:68793666-chr16:72915551_1	CDH1	intronic
RM79	chr17	31350606	BND	pbsv.BND.chr17:3,1350606-chr1:248935729_1	NF1	intronic
RM80	chr6	151943280	BND	pbsv.BND.chr6:151943280-chr17:38647479_1	ESR1	intronic

## Discussion

A previous study demonstrates that PacBio long reads could detect over 20,000 SVs in a typical whole human genome (Chaisson et al., 2015). However, whole-genome third-generation sequencing is rather expensive and limits its clinical applications. To address this issue, we applied to the best of our knowledge the first clinical TGS panel using the PacBio HiFi platform to breast cancer samples. We conducted a comprehensive analysis on structural variations across 28 breast cancer-related genes through long-read genomic and transcriptomic sequencing of paired breast cancer tissue and blood. We compared the genome of tumor and paratumor tissue in 19 breast cancer patients to identify somatic SVs. We also compared the genome of white-blood-cell from 19 breast cancer patients and seven healthy controls to identify possible pathogenic SVs. Our results suggested that germline and somatic SVs were common in the selected genes among breast cancer patients, though the majority of them occurred in the non-exonic region. We also identified a potential hotspot region for somatic SVs. Taking together, our results demonstrated that SVs are potentially important in the tumorigenesis of breast cancer. Indeed, the International Cancer Genome Consortium (ICGC) previously showed that driver SVs are more prevalent than point mutations in breast adenocarcinomas (6.4 SVs compared with 2.2 point mutations on average) (Consortium, 2020).

The traditional NGS platforms have a poor mapping to repetitive elements, including tandem repeats and interspersed repeats, which has made a substantial fraction of most genomes inaccessible and limited its ability to detect SVs (Sedlazeck et al., 2018). One a representative type of interspersed repeats is Alu element which accounted for 11% of the human genome sequences on average, it belongs to a class of retroelements termed short interspersed nuclear elements (SINEs) and often causes SVs through homologous recombination (Doronina et al., 2021). An important reason that we developed the 28-gene TGS panel for illuminating the full landscape of SVs in breast cancer is to overcome the limitations of NGS in detecting SVs around repetitive elements. The repetitive elements are abundant in the 28-gene panel which contains most of the breast cancer-related genes, for instance, the *BRCA1* gene has around 40% of Alu family repetitive elements in its DNA sequences (Sobczak and Krzyzosiak, 2002; Ewald et al., 2009).

In this paper, by acquiring paired blood, paratumor and tumor tissue from patients, we delineated germline and somatic mutations which were both reported to be responsible for carcinogenesis. Interestingly, we found a potential somatic SV hotspot in the AT-rich region of *ERBB2* gene. Although this region does not belong to interspersed repeats which often causes SVs through homologous recombination, there are proofs in previous studies that SV hotspots could exist in regions other than SINE elements and DNA transposons (Lin and Gokcumen, 2019). Hence, our method of fine-scale characterization of genomic structural variations using TGS holds great potential to elucidate the full landscape of SV in breast cancer.

We have also systematically examined the paratumor tissues which was used as control samples to identify somatic mutations in tumor. During the process of carcinogenesis, somatic mutations continuously accumulated within the tumor tissue, turning the genomic structure different from its surrounding paratumor tissues (Shoshani et al., 2021). It is essential to figure out to which extent the paratumor is different from the blood and tumor. We have shown that most SVs were the same in both blood and paratumor tissues, but different from those in the breast cancer tissues. This is in accordance with a previous study that demonstrated paratumor and tumor show very different occurrence patterns in copy number variations (Hu et al., 2018).

Our 28-gene TGS panel also showed great promise in identify casual SVs of breast cancer. *NF1* is one of the 12 breast cancer predisposition genes identified to date, however, virtually all previous studies have focused on evaluating breast cancer risk associated with putative pathogenic SNVs and small InDels (Chen et al., 2021; Hu et al., 2021). We have successfully identified two exonic SVs in two breast tumor tissues, which proves that our TGS panel is useful for detecting cancer-related SVs. Moreover, our TGS panel is robust in identifying SVs, as indicated by the concordance of most fusion genes identified by long-read genomic and transcriptomic sequencing.

Nevertheless, there are still a few hurdles that remain to address. The first one would be to study more cases. The main roadblock to this purpose was the high cost of TGS at the time of writing; hopefully, as the TGS market continues to grow, the price could eventually drop to a more reasonable level. The second one would be to substantiate the hypothesis of LOH occurrence in some SV regions. The challenge lies in the read-length limitation of the contemporary panel sequencing. We believe the advance of technologies would breakthrough in this section, as we have seen the industry pushing the read-length longer every few years. Last but not least, we plan to further evaluate the effectiveness of our TGS analysis pipeline by running it against public datasets side by side with most of the analysis methods available in the market.

## Conclusion

In conclusion, we found that somatic SVs were abundant in the breast cancer genome, which suggests that they may play an important role in the process of tumorigenesis and cancer development. This is especially important for breast cancer, since the pan-cancer studies conducted by ICGC found that the driver SVs was most evidently prevalent in breast cancer compared to driver point mutations (Consortium, 2020). Taking together, our clinical TGS panel shown here is an accurate and robust method to detect SVs in breast cancer, which is both important for breast cancer research and holds great potential for further clinical application.

## Abbreviations

CCS, Circular Consensus Sequence; DCIS, Ductal Carcinoma *In Situ*; HRR, Homologous Recombination Repair; ICGC, International Cancer Genome Consortium; NGS, next-generation sequencing; ONT, Oxford Nanopore Technologies; RIN, RNA Integrity Number; SINE, Short Interspersed Nuclear Element; SMRT, Single-Molecule Real-Time; SNV, Single Nucleotide Variation; SDS, Sodium Dodecyl Sulphate; SV, Structural Variation; TGS, Third-Generation Sequencing; TNBC, Triple Negative Breast Cancer; UTR, UnTranslated Region.

## Data Availability

The datasets presented in this study can be found in online repositories. The name of the repository and accession number can be found below: National Genomics Data Center (https://ngdc.cncb.ac.cn/); PRJCA008780.

## Abbreviations

CCS, Circular Consensus Sequence; DCIS, Ductal Carcinoma In Situ; HRR, Homologous Recombination Repair; ICGC, International Cancer Genome Consortium; NGS, next-generation sequencing; ONT, Oxford Nanopore Technologies; RIN, RNA Integrity Number; SINE, Short Interspersed Nuclear Element; SMRT, Single-Molecule Real-Time; SNV, Single Nucleotide Variation; SDS, Sodium Dodecyl Sulphate; SV, Structural Variation; TGS, Third-Generation Sequencing; TNBC, Triple Negative Breast Cancer; UTR, UnTranslated Region.
